# The Impact of Preventive Protocols on Oral Health Outcomes in Cancer Patients Undergoing Chemotherapy or Radiotherapy: A Systematic Review and Meta-Analysis

**DOI:** 10.3390/diseases13060186

**Published:** 2025-06-16

**Authors:** Carol Moussa, Laurent Estrade, Jeremy Glomet, Gael Y. Rochefort, Frédéric Denis, Maha H. Daou

**Affiliations:** 1 Faculty of Dentistry, University of Tours, 37032 Tours, France; gael.rochefort@univ-tours.fr (G.Y.R.); frederic.denis@univ-tours.fr (F.D.); maha.daou@univ-tours.fr (M.H.D.); 2 Division of Education, Ethics, Health, Faculty of Medicine, University of Tours, 37044 Tours, France; 3 Department of Medicine and Bucco-Dental Surgery, Tours University Hospital, 37044 Tours, France; l.estrade@chu-tours.fr (L.E.); jeremy.glomet@univ-tours.fr (J.G.); 4 INSERM, Imaging Brain & Neuropsychiatry iBraiN U1253, 37032 Tours, France; 5 Department of Pediatric Dentistry, Faculty of Dental Medicine, Saint Joseph University, Beirut 1107 2180, Lebanon; 6 Craniofacial Research Laboratory, Division of Biomaterials, Saint Joseph University, Beirut 1107 2180, Lebanon

**Keywords:** preventive dentistry, oral health, cancer patients, chemotherapy, radiotherapy, fluoride, oral hygiene

## Abstract

**Background/Objectives**: Cancer patients undergoing chemotherapy (CT) or radiotherapy (RT) are at increased risk of oral complications. Preventive dental care has been proposed to mitigate these risks, yet its effectiveness is not sufficiently evaluated. This systematic review and meta-analysis aimed to assess the impact of preventive oral health interventions on key clinical outcomes in oncology patients. **Methods**: A systematic search of MEDLINE, Embase, and CENTRAL databases was conducted (March 2025), adhering to PRISMA guidelines with a PROSPERO-registered protocol (CRD 420251006799). Eligible studies included randomized trials, cohort studies, and pre–post intervention studies evaluating preventive dental care in patients receiving CT or RT. The outcomes included gingival index (GI), dental caries (DMFT), plaque levels, and periodontal health. Meta-analyses were performed on GI and DMFT outcomes using random-effects models. **Results**: Eleven studies were included in the qualitative synthesis and four in the meta-analyses. Preventive interventions, such as fluoride applications, oral hygiene education, and regular professional cleanings, were associated with stabilization or improvement of gingival health. The pooled estimate for GI showed no significant deterioration over time (MD = −0.05, 95% CI: −0.34 to 0.24, *p* = 0.72). For DMFT, a slight but significant increase was observed (MD = 1.07, 95% CI: 0.08 to 2.05, *p* = 0.03), suggesting a continued risk of caries despite intervention. **Conclusions**: Preventive dental care interventions appear to support the maintenance of gingival health in cancer patients undergoing CT or RT. However, despite these interventions, a slight increase in dental caries was still observed, indicating that preventive strategies may not fully eliminate the risk of caries. These findings highlight the importance of sustained and individualized oral health programs as part of comprehensive oncology care. Future studies using standardized protocols and longer follow-up periods are needed to better evaluate their long-term effectiveness across diverse cancer populations.

## 1. Introduction

Cancer remains a leading cause of morbidity and mortality worldwide, with treatment modalities such as chemotherapy (CT) and radiotherapy (RT) playing a vital role in improving prognosis [1]. However, these therapies often result in adverse effects on the oral cavity, including mucositis, xerostomia, infections, and increased risk of dental caries and periodontal disease [2]. Oral complications, such as dental caries, pulp infections, dental abscesses, or osteonecrosis, can significantly impair nutritional intake, speech, and overall quality of life and may even necessitate interruption of cancer therapy [3].

Beyond these local effects, cancer therapies, particularly CT, suppress immune function by targeting rapidly dividing hematopoietic cells [4]. This immunosuppression compromises host defense mechanisms, especially in the oral cavity, where mucosal damage and microbial imbalance create ideal conditions for opportunistic infections [4,5]. As a result, dental infections in these patients may progress to systemic complications such as bacteremia or sepsis [6,7,8].

In this context, the early integration of preventive dental care into oncology treatment planning has the potential to attenuate these complications [9]. By assessing oral health status prior to therapy initiation and implementing individualized care strategies, dental professionals can support systemic treatment while improving patients’ overall well-being [10]. Several international guidelines, such as those from the Multinational Association of Supportive Care in Cancer/International Society of Oral Oncology (MASCC/ISOO) and the European Society for Blood and Marrow Transplantation (EBMT), recommend integrating preventive oral care into standard cancer management protocols to reduce complications and maintain oral function during therapy [6,11].

Traditionally, oral healthcare in oncology has focused on the management of established disease, making treating infections, performing extractions, and restoring decayed teeth more complex. Unfortunately, this curative approach often comes too late [7,12,13,14]. The cumulative effects of oral mucosal damage, reduced salivary flow, microbial imbalance, and immune fragility highlight the urgent need for preventive dental care strategies in cancer patients.

Preventive measures, such as fluoride therapy, oral hygiene education, and comprehensive oral care programs, have been proposed to reduce the incidence and severity of oral complications [6]. Early intervention and consistent monitoring can lessen the severity of complications, ultimately enhancing both oral and systemic health outcomes for patients.

Despite the increasing recognition of the importance of oral health management in oncology, preventive strategies are not always implemented enough in healthcare facilities. Furthermore, the effectiveness of various interventions varies, highlighting the need for a systematic evaluation of the available evidence.

While previous reviews have largely concentrated on managing dental diseases and infection control in cancer patients [12,15], our study aims to assess the impact of preventive oral health interventions on key clinical outcomes in patients undergoing cancer treatment. By focusing on parameters such as plaque control, gingival inflammation, caries incidence, and periodontal health, this review integrates findings from studies employing various preventive strategies in oncology populations.

## 2. Materials and Methods

### 2.1. Data Sources

This study adheres to the Cochrane Guidelines and follows the Preferred Reporting Items for Systematic Reviews and Meta-Analyses (PRISMA) framework [16]. The protocol was prospectively registered in the PROSPERO database under the registration number CRD 420251 006799.

A review protocol was developed prior to the study, guided by the PICO framework. The central research question was defined as follows: in cancer patients undergoing CT or RT (P), does the implementation of preventive dental care interventions (I) lead to improved oral health outcomes (O) compared to those receiving standard care or no preventive intervention (C)?

### 2.2. Search Strategy

**Figure 1:**
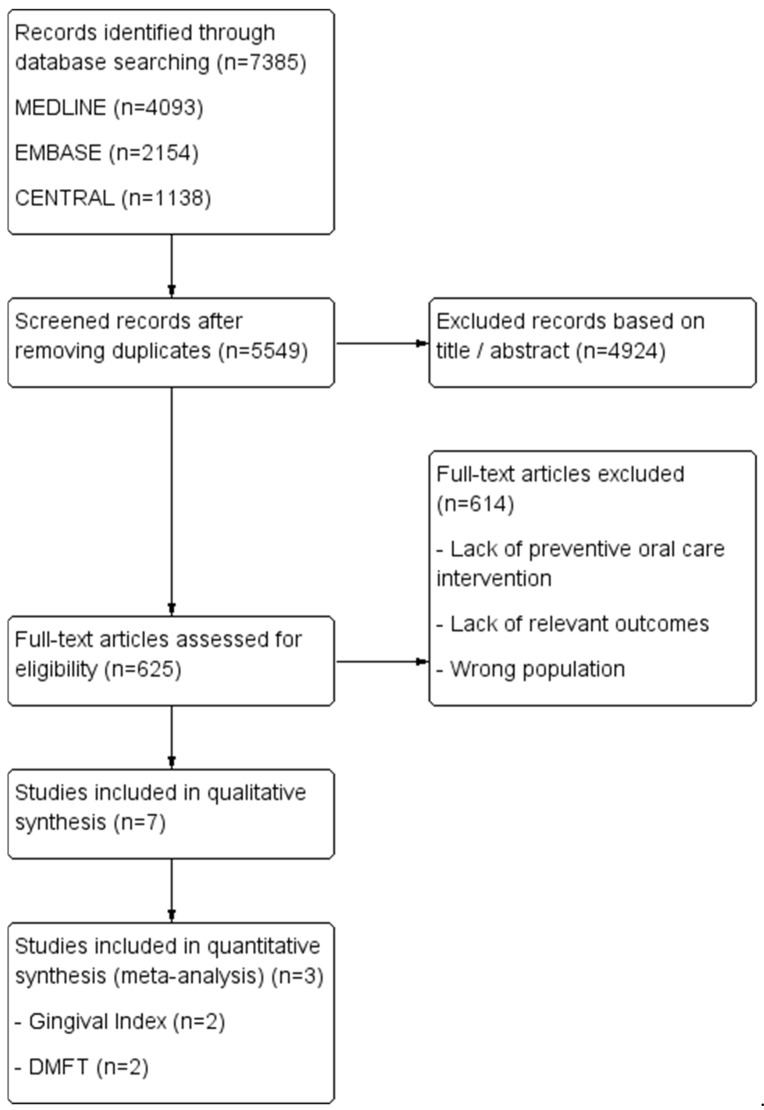
Study flow diagram of the inclusion process.

A comprehensive literature search was conducted on 7 March 2025, across MEDLINE, Embase, and the Cochrane Central Register of Controlled Trials (CENTRAL). Two independent reviewers (C.M. and M.H.D.) performed the search using predefined search strategies, detailed in Table 1. The overall study selection and inclusion process is illustrated in the flow diagram (Figure 1).

### 2.3. Selection Criteria

The article selection process was carried out independently by two reviewers (C.M. and M.H.D.), who screened titles and abstracts to identify studies meeting the eligibility criteria. Studies were included if they reported original research evaluating the effectiveness of preventive oral care interventions in oncology patients undergoing CT or RT. The intervention had to be implemented before or at the initiation of cancer therapy, and outcomes had to be assessed during or after treatment, allowing for a temporal evaluation of its effectiveness.

Eligible study designs included randomized controlled trials, cohort studies (prospective or retrospective), longitudinal studies, cross-sectional studies, quasi-experimental studies (with or without control groups), and observational pre–post intervention studies.

In recognition of the limited availability of high-level evidence in this area, well-documented pre–post intervention studies without control groups were also included when they assessed relevant clinical outcomes such as mucositis severity, plaque index, or the incidence of caries. Studies that focused exclusively on disease prevalence or lacked a clearly defined intervention were excluded.

To ensure contemporary relevance, the search was limited to studies published since 2014 and to articles available in English or French.

The following types of publications were excluded: case reports, literature review, letters to the editor, expert opinions, conference abstracts, case series, and pilot studies. All articles that met the initial inclusion criteria were retrieved in full and underwent full-text review to confirm their final eligibility for inclusion.

### 2.4. Data Extraction and Analysis

All studies meeting the inclusion criteria were independently screened and assessed in full by two reviewers (C.M. and M.H.D.). Any discrepancies arising during the selection process were resolved through discussion and consensus prior to proceeding with data extraction.

For studies eligible for statistical synthesis, Review Manager (RevMan) version 5.4.1 (Cochrane Collaboration, Oxford, UK) was used. Continuous variables were analyzed using the inverse variance method and reported as weighted mean differences (WMDs) with 95% confidence intervals (CIs).

Statistical significance was set at *p* < 0.05. Heterogeneity was evaluated using the Chi-square test (with N − 1 degrees of freedom) and quantified with the I^2^ statistic. A random-effects model was applied to account for between-study variability. All statistical analyses were performed using RevMan version 5.4.1 [17].

For the risk of bias assessment, each study was independently assessed by the two reviewers and disagreements were resolved by discussion. Studies were categorized as having a low, moderate, or high risk of bias based on these assessments.

### 2.5. Classification of Study Designs

The classification of study designs was based on a detailed review of the methodological approach described in each article. Studies were categorized according to standard epidemiological definitions. Pre–post observational studies were defined as those in which outcomes were measured before and after an intervention in a single group without randomization or a control group. Quasi-experimental studies were identified as those employing a pre–post design with structured interventions but lacking random allocation. Cohort studies, whether prospective or retrospective, were distinguished by the presence of clearly defined groups followed over time to assess outcomes, with or without a control group. Cross-sectional studies were defined as those assessing exposure and outcomes at a single point in time.

### 2.6. Meta-Analysis

Two separate meta-analyses were conducted to evaluate changes in oral health outcomes—specifically the gingival index (GI) and Decayed, Missing, and Filled Teeth Index (DMFT)—among cancer patients, following preventive dental interventions. Studies were eligible for inclusion if they reported both pre- and post-intervention mean values and standard deviations (SDs) for the relevant oral health index.

When follow-up values were available for a single group only, change scores were calculated and entered the analysis using a dummy comparator group (mean = 0, SD = 1, same sample size) to allow inclusion in Review Manager (RevMan). For studies with true comparison groups, direct between-group analyses were performed.

**Figure 2:**
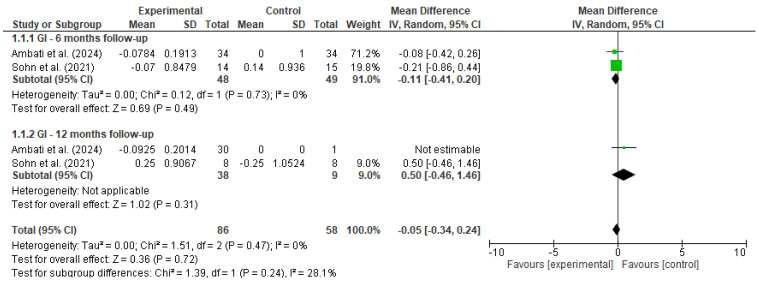
Forest plot for GI [18,19].

**Figure 3:**
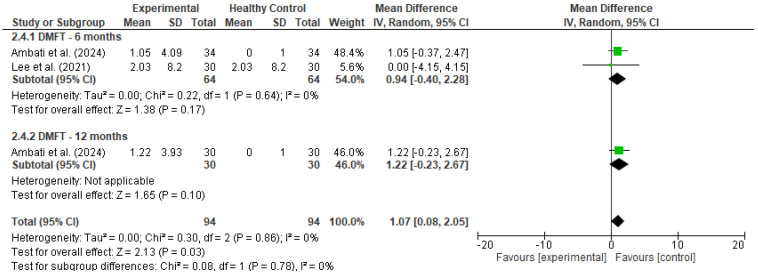
Forest plot for DMFT [19,20].

Pooled estimates were calculated using a random-effects model to account for methodological and clinical heterogeneity across studies, even when statistical heterogeneity was low [17]. Outcomes were stratified by follow-up duration (e.g., 6 and 12 months), and heterogeneity was assessed using the I^2^ statistic. Forest plots (Figure 2 and Figure 3) were generated for each outcome to visually represent effect sizes and confidence intervals.

## 3. Results

### 3.1. Study Selection and Screening Results

A total of 7385 articles were initially identified from the database search. After removing duplicates, 5549 articles remained for title and abstract screening, leading to the exclusion of 4924 articles. Subsequently, 625 articles underwent full-text review, of which 11 studies met the criteria for inclusion into the qualitative synthesis, while 4 studies were eligible for inclusion in the meta-analyses.

### 3.2. Characteristics of the Included Studies for Qualitative Synthesis

The characteristics of the included studies, evaluating the effectiveness of preventive oral care programs in oncology patients, are summarized in Table 2. The extracted data includes study design, sample size, type of intervention, outcome measures assessed, and key findings reported in each study.

### 3.3. Risk of Bias

Risk of bias was assessed according to the study design using standardized tools. For non-randomized comparative studies (cohort, case–control, quasi-experimental), the Risk Of Bias In Non-randomized Studies-of Interventions (ROBINS-I) tool was used [25]. Pre–post studies without control groups were assessed using the National Institutes of Health (NIH) Quality Assessment Tool for Pre–Post Studies [26], while cross-sectional studies were evaluated with the Appraisal tool for Cross-Sectional Studies (AXIS) tool [27,28] (Table 3).

### 3.4. Statistical Findings in Selected Articles

Table 4 presents a summary of the key statistical outcomes reported in the selected studies. The table highlights changes in oral health indicators—gingival index (GI), plaque index (PI), and Decayed, Missing, and Filled Teeth Index (DMFT)—before and after the implementation of preventive dental interventions. These findings provide insight into the effectiveness of various oral care strategies.

### 3.5. Results of Meta-Analyses

To facilitate comparison across studies, a summary table (Table 5) presents only the clinical outcomes selected for quantitative synthesis. These include the gingival index (GI), plaque index (PI), and Decayed, Missing, and Filled Teeth Index (DMFT), which were consistently reported across the included studies and align with the primary objectives of this review.

#### 3.5.1. Gingival Index (GI)

A meta-analysis was performed to evaluate the effectiveness of preventive dental interventions in maintaining gingival health over time, as measured by changes in the gingival index (GI). Two studies—Ambati et al. [18] and Sohn et al. [19]—provided pre- and post-intervention GI data suitable for calculating within-group change scores.

In Sohn et al.’s study, comparisons were conducted between a vulnerable population and a healthy control group, while Ambati et al. included only a single intervention group. To enable the inclusion of Ambati et al.’s study in the pooled analysis using Review Manager (RevMan), a dummy comparator group (mean = 0, SD = 1, same sample size) was introduced.

Change scores and corresponding standard deviations were estimated using a correlation coefficient of 0.5 between baseline and follow-up measurements. A random-effects model was applied to account for both clinical and methodological heterogeneity between studies.

At the 6-month follow-up, the pooled mean difference was −0.11 (95% CI: −0.41 to 0.20, *p* = 0.49), with no statistical heterogeneity (I^2^ = 0%). At 12 months, only Sohn et al. provided comparative data between groups, yielding a non-significant mean difference of 0.50 (95% CI: −0.46 to 1.46, *p* = 0.31). When all timepoints were considered, the overall pooled estimate indicated no significant change in gingival condition (MD = −0.05, 95% CI: −0.34 to 0.24, *p* = 0.72), with low heterogeneity (I^2^ = 0%).

These findings suggest that, over a 6 to 12-month period, the implemented preventive interventions were effective in maintaining gingival health, with no evidence of deterioration in gingival status among the studied populations.

#### 3.5.2. DMFT

A meta-analysis was conducted to evaluate changes in the DMFT index among cancer patients following the implementation of preventive dental interventions. Data were pooled from two studies: Ambati et al. [18], which reported within-group changes over 6 and 12 months, and Lee et al. [20], which compared cancer patients who received preventive care to those who did not. For Ambati’s study, a dummy control group was used to allow within-group change analysis; in Lee’s study, the true comparison between experimental (with prevention) and control (without prevention) groups was utilized.

At 6 months, the pooled mean difference was 0.94 (95% CI: −0.40 to 2.28, *p* = 0.17), with no statistical heterogeneity (I^2^ = 0%). At 12 months, the single included study (Ambati) showed a mean DMFT increase of 1.22 (95% CI: −0.23 to 2.67, *p* = 0.10). As this estimate is derived from a single study, it is presented for descriptive purposes only and should not be interpreted as a standalone sub-analysis. The pooled analysis remains the primary focus for interpretation. When all timepoints were combined, the overall pooled mean difference was 1.07 (95% CI: 0.08 to 2.05, *p* = 0.03), indicating a statistically significant, though modest, increase in DMFT scores over time.

These findings suggest that despite the implementation of preventive interventions, there may still be a slight progression in dental caries in cancer patients over time. However, the low heterogeneity (I^2^ = 0%) and the confidence intervals spanning both sides of the null in the subgroup analyses warrant cautious interpretation.

## 4. Discussion

This systematic review examined the impact of preventive oral care programs on oral health outcomes in cancer patients, with the primary objective of evaluating the effectiveness of these interventions across the populations. Despite considerable variability in study designs, outcome measures, and patient groups, all included studies shared a common goal: to assess how preventive dental strategies influence oral health in individuals undergoing cancer treatment.

Several studies assessed similar oral health parameters, including plaque index, gingival index (GI), history of caries, and periodontal pocket depth. However, variability in intervention protocols, follow-up duration, and measurement methodologies limited the feasibility of conducting large-scale pooled analyses across all outcomes. As a result, two separate meta-analyses were performed, focusing specifically on GI and DMFT outcomes among cancer patients receiving preventive dental care. Despite the limited number of eligible studies, the findings were consistent and suggest that structured preventive interventions may contribute to the maintenance of gingival and dental health over time in this vulnerable population.

Plaque index was among the most reported outcomes across the included studies. Both Lee [20] and Sohn [19] conducted longitudinal assessments, with Sohn extending the follow-up period to twelve months. In contrast, Bertl et al. [21] examined plaque levels in relation to the frequency of professional tooth cleaning, reporting a clear association between more frequent care and lower plaque scores. Morais et al. [23] also evaluated plaque accumulation within a structured preventive care protocol, observing consistent reductions over time, despite the absence of a control group.

Although differences in study design, follow-up duration, and plaque measurement methods limit the possibility of direct comparisons, the overall findings support the effectiveness of regular preventive interventions in reducing plaque accumulation during and after cancer treatment. In particular, Morais et al. highlighted the adjunctive role of PBMT in reducing the severity of mucositis, demonstrating its potential value within multi-component preventive protocols [23].

Periodontal pocket depth was another commonly assessed parameter, evaluated across three studies, each employing a distinct methodological approach. Sohn et al. [19] measured clinical pocket depth at multiple timepoints in both healthy individuals and cancer patients, allowing for a longitudinal assessment of preventive care outcomes. Bertl et al. [21] analyzed the number of teeth presenting with deep periodontal pockets in relation to oral hygiene behaviors, while Morais et al. [23] used a Periodontal screening and recording (PSR) system to track localized periodontal changes during radiotherapy.

Despite differences in measurement techniques and study design, all three studies reported improvements in periodontal parameters following the implementation of preventive interventions. These findings suggest a consistently positive effect of preventive care on periodontal health, even in the context of methodological heterogeneity.

The gingival index (GI) was assessed in two studies—Ambati et al. [18] and Sohn et al. [19]—yielding notably divergent results. Ambati reported a marked reduction in gingival inflammation over time among pediatric patients receiving preventive care, indicating a strong positive response to the intervention. In contrast, Sohn’s adult cohort exhibited stable GI scores over a 12-month period, with no statistically significant changes.

This discrepancy may be attributed to differences in age groups, cancer types, and baseline oral health status, underscoring the need to tailor preventive strategies to the specific characteristics of different oncology subpopulations.

History of caries, most often assessed using the Decayed, Missing, and Filled Teeth (DMFT) Index, was reported across several studies. Ambati et al. [18] observed a progressive increase in DMFT scores among pediatric patients, despite the implementation of preventive measures, highlighting the ongoing susceptibility of this population. In contrast, Lee et al. [20] reported stable DMFT scores in the group receiving preventive care, whereas the group without such interventions showed a significant increase, indicating a potential protective effect.

Further supporting this observation, Frydrych et al. [24] demonstrated that patients who adhered to structured preventive strategies—such as dietary counseling, fluoride application, and oral hygiene instruction—had a significantly lower incidence of caries during and after cancer treatment. While Ambati et al. provided 12-month DMFT data that was included in the forest plot, this estimate reflects a single-study observation and was not intended as a standalone sub-analysis. Its inclusion was primarily descriptive, and we have revised the text accordingly to avoid overinterpretation. Collectively, these findings suggest that comprehensive and consistent preventive oral and dental care may play a critical role in mitigating the risk of caries and oral disease in oncology patients.

Rather than relying solely on the DMFT index, some studies focused specifically on the number of decayed teeth as a direct measure of caries activity. Sohn et al. [19] conducted a longitudinal assessment in cancer patients, observing that regular follow-up and sustained oral hygiene practices were associated with the stabilization of caries progression during therapy. Similarly, Bertl et al. [12], in a retrospective analysis, reported a significantly higher number of decayed teeth among patients who received professional dental cleaning less than once per year compared to those who underwent more frequent preventive care. Although Bertl et al. did not assess a pre-treatment intervention, it was included in the qualitative synthesis due to its focus on the relationship between preventive dental behaviors and post-treatment oral health outcomes.

These findings further underscore the importance of regular preventive interventions in limiting the development or progression of active carious lesions in patients undergoing cancer treatment.

In addition to the core oral health outcomes, several studies investigated complementary parameters that provide a broader understanding of oral health challenges in oncology patients. Bertl et al. [21] assessed the presence of periodontitis as a clinical diagnosis, offering a more holistic perspective on periodontal health beyond standard indices. Morais et al. [23] used the Oral Assessment Guide (OAG) to monitor general oral complications during radiotherapy. Meanwhile, Frydrych et al. [24] evaluated the need for dental extractions following cancer treatment and found a strong association with non-compliance to preventive protocols, which included adherence to a non-cariogenic diet, regular dental visits, proper oral hygiene, and daily use of high-fluoride toothpaste.

Although methodological differences limit direct comparisons across studies, the collective findings underscore the multidimensional impact of cancer therapy on oral health and the critical role of preventive strategies in mitigating this burden. Importantly, cancer treatments induce a state of immunosuppression that heightens susceptibility to oral infections, including dental caries and periodontal diseases, which are primarily infectious in nature [29,30,31].

Differences in the reported effectiveness of preventive interventions may reflect underlying heterogeneity in patient populations. Variability in age, cancer type, baseline oral health, and socioeconomic status, as well as the type of anticancer agents administered, the stage of disease, and the general condition of the patient, can all influence oral health vulnerability and treatment response. These factors underscore the importance of tailoring preventive dental strategies to the specific needs of distinct oncology subpopulations.

The reviewed studies demonstrate that timely and structured interventions, including pre-treatment oral health assessments, patient education, dietary counseling, professional prophylaxis, daily use of high-fluoride toothpaste, and regular dental follow-up, can effectively reduce complications and help preserve oral function during and after cancer therapy. To further strengthen the evidence base, future research should adopt standardized intervention protocols, define core outcome measures, and incorporate longer follow-up periods to assess the sustained impact.

## 5. Limitations

The principal limitation of this review is the substantial heterogeneity among the included studies. Variations in study design, patient populations, cancer types, follow-up durations, and outcome assessment methods limited the ability to perform direct comparisons and constrained the feasibility of conducting meta-analyses beyond a few specific outcomes. Notably, the site and type of cancer, particularly distinctions between head and neck versus systemic or non-craniofacial malignancies, likely influenced the oral impact of treatments and contributed to the heterogeneity of findings. To provide greater clarity on these variations, Supplementary Table S1 presents a detailed overview of key intervention characteristics across studies, including population type, hygiene instruction, fluoride use, PBMT application, cancer context, and evaluation timepoints.

Although multiple studies investigated comparable oral health parameters, the use of inconsistent definitions and diverse evaluation tools hindered standardized interpretation and synthesis. To better contextualize these variations, we have included a Supplementary Table S1 detailing cancer site, treatment modality, and preventive strategies for each study.

Despite its limitations, this review has several notable strengths. First, it provides a comprehensive and up-to-date synthesis of the available evidence on preventive dental care in oncology settings, an area that remains underrepresented in the literature. By focusing on both quantitative outcomes and clinical relevance, the review offers valuable insights into the potential benefits of structured oral and dental care interventions for cancer patients and highlights areas in need of further investigation.

## 6. Conclusions

Preventive dental care interventions are essential for preserving oral health in cancer patients undergoing CT or RT. This systematic review and meta-analyses demonstrate consistent evidence that structured and timely oral health protocols can help stabilize clinical outcomes and mitigate treatment-related complications. Despite variability in study design, population characteristics, and outcome assessment methods, the overarching trend supports the integration of tailored preventive strategies as a fundamental component of comprehensive oncology care. Moving forward, future research should focus on developing standardized intervention protocols, comparing outcome measures, and conducting rigorous, large-scale studies to enable meaningful comparisons and generate high-quality evidence for clinical guideline development

## Figures and Tables

**Table 1:** Search strings.

Database	Search String
**Medline**	((“Oral Health” [Mesh] OR “Dental Care” [Mesh] OR “Preventive Dentistry” [Mesh] OR “Mouth Diseases/prevention and control” [Mesh] OR “Fluorides” [Mesh] OR “Fluoride Therapy” [Mesh]) AND (“Neoplasms”[Mesh] OR “Chemotherapy” [Mesh] OR “Radiotherapy” [Mesh] OR “Cancer Patients” [Mesh] OR “Oncology Patients” [Mesh]))
**Embase**	(‘oral health’/exp OR ‘dental care’/exp OR ‘preventive dentistry’/exp OR ‘mouth disease prevention’/exp OR ‘fluoride’/exp OR ‘fluoride therapy’/exp) AND (‘neoplasm’/exp OR ‘cancer patient’/exp OR ‘chemotherapy’/exp OR ‘radiotherapy’/exp)
**Central**	(“Preventive Dentistry” OR “Dental Prophylaxis” OR “Fluoride Therapy” OR “Oral Hygiene” OR “Mouth Rinses” OR “Fluoride”) AND (“Cancer Patients” OR “Neoplasms” OR “Oncology”) AND (“Chemotherapy” OR “Radiotherapy” OR “Antineoplastic Agents”) AND (“Oral Health” OR “Oral Mucositis” OR “Xerostomia” OR “Dental Care”)

**Table 2:** Characteristics of included studies.

Study	Intervention	Outcome Measures	Key Findings
**Ambati et al. [18]**	Oral hygiene education, fluoride varnish, SDF, sealants, mouthwash, and topical analgesics	Mucositis severity, gingival index, oral hygiene index, dental caries (DMFT)	Oral mucositis severity decreased over time; however, caries remained prevalent, underscoring the need for continued preventive care.
**Lee et al. [20]**	Oral health education (4 sessions), fluoride varnish, daily fluoride mouthrinse	Plaque index, salivary flow rate, bleeding on probing, dental caries, quality of life	Oral health and quality of life improved significantly following preventive care; multidisciplinary collaboration recommended.
**Bertl et al. [21]**	Self-reported preventive behaviors: dental visits, hygiene practices, mouthwash use	Dental caries (DMFT), periodontal disease, probing depth, bleeding on probing	Low compliance with preventive behaviors was associated with poorer oral health; highlights the gap in post-treatment dental care.
**Amin et al. [22]**	Oral hygiene instruction over six weeks	Simplified Oral Hygiene Index (OHI-S), Community Periodontal Index (CPI)	Significant improvement in oral hygiene in 26% of participants; patient education shown to be effective.
**Morais et al. [23]**	Preventive oral care including hygiene, restorations, hydration, PBMT	Oral mucositis severity, pain, dysphagia, quality of life, RT interruption	Preventive program combined with PBMT effectively reduced mucositis and improved treatment continuity.
**Sohn et al. [19]**	Professional hygiene care weekly during RT and quarterly post-RT, fluoride varnish	Plaque index, gingival index, dental caries, periodontal attachment loss	Regular hygiene care was effective in maintaining oral health over 12 months post-radiotherapy.
**Frydrych et al. [24]**	Dietary advice, dental care, hygiene instruction, fluoride use over 12 months	Compliance with care protocols and the development of caries	Non-compliance with preventive strategies was significantly associated with increased risk of caries.

**Table 3:** Risk of bias summary.

Study	Design	Risk of Bias Tool	Overall Risk of Bias
Ambati et al. [18]	Prospective observational pre–post study	NIH Pre–Post Tool	Moderate
Lee et al. [20]	Quasi-experimental (pre–post, no control group)	ROBINS-I	Moderate
Bertl et al. [21]	Cross-sectional	AXIS	Moderate
Amin et al. [22]	Prospective observational pre–post study	NIH Pre–Post Tool	Moderate
Morais et al. [23]	Prospective cohort study with pre–post assessments	NIH Pre–Post Tool	Moderate
Sohn et al. [19]	Prospective cohort study	ROBINS-I	Low
Frydrych et al. [24]	Retrospective observational pre–post study	NIH Pre–Post Tool	Moderate

**Table 4:** Oral health outcomes in the selected articles.

Study	Outcome Measure	Reported Results
**Ambati et al. [18]**	Oral mucositis (WHO scale)	Baseline (*n* = 44): Grade 0: 25 (56.8%), I: 9 (20.5%), II: 4 (9.1%), III: 4 (9.1%), IV: 2 (4.5%) First follow-up (3–6 months) (*n* = 34): Grade 0: 19 (55.88%), I: 8 (23.52%), II: 5 (14.70%), III: 1 (2.95%), IV: 1 (2.95%) Second follow-up (9–12 months) (*n* = 30): Grade 0: 25 (83.3%), I: 4 (13.3%), III: 1 (3.3%)
	Gingival Index (mean ± SD)	Baseline (*n* = 44): 0.0983 ± 0.21549 First follow-up (*n* = 34): 0.0199 ± 0.06565 Second follow-up (*n* = 30): 0.0058 ± 0.03195
	Oral hygiene index; simplified (DI-S) (mean ± SD)	Baseline (*n* = 44): 1.5820 ± 1.13729 First follow-up (*n* = 34): 1.3350 ± 0.85867 Second follow-up (*n* = 30): 1.6213 ± 1.14416
	DMFT (mean ± SD)	Baseline (*n* = 44): 3.9773 ± 3.92659 First follow-up (*n* = 34): 5.0294 ± 4.23181 Second follow-up (*n* = 30): 5.2000 ± 3.92516
**Lee et al. [20]**	DMFT (mean ± SD)	Experimental group (*n* = 31): before: 5.45 ± 5.64; after 6 months: 5.45 ± 5.77; *p* = 1.000 Control group (no preventive dental care) (*n* = 30): before: 6.37 ± 8.12; after 6 months: 8.40 ± 8.27; *p* = 0.006 *
	Plaque score (O’Leary percentage) (mean ± SD)	Experimental group (*n* = 31): before: 24.70 ± 22.23; after 6 months: 5.90 ± 5.00; *p* < 0.001 * Control group (*n* = 30): before: 15.74 ± 12.25; after 6 months: 21.52 ± 20.61; *p* = 0.113
	Bleeding on probing (mean ± SD)	Experimental group (*n* = 31): before: 8.41 ± 12.32; after 6 months: 2.83 ± 6.64; *p* = 0.004 * Control group (*n* = 30): before: 8.85 ±10.56; after 6 months: 10.41 ± 13.25; *p* = 0.388
	Salivary flowrate (mL/min, mean ± SD)	Experimental group (*n* = 31): before: 1.18 ± 0.56; after 6 months: 0.88 ± 0.41; *p* = 0.027 * Control group (*n* = 30): before: 1.29 ± 0.72; after 6 months: 0.66 ± 0.42; *p* = 0.001 *
**Bertl et al. [21]**	Presence of periodontitis	Dental consultation prior to treatment: No = 19/22 present, Yes = 17/24 present Last dental check-up: <12 months = 19/28 present, ≥12 months = 17/18 present Frequency of professional tooth cleaning: <1/year = 22/27 present, ≥1/year = 14/19 present
	≥1 Tooth with probing pocket depth (PD) ≥ 5 mm	Dental consultation prior to treatment: no = 10/22 present, yes = 9/24 present Last dental check-up: <12 months = 13/28 present, ≥12 months = 6/18 present Frequency of professional tooth cleaning: <1/year = 10/27 present, ≥1/year = 9/19 present
	≥1 Tooth with caries	Dental consultation prior to treatment: no = 17/20 present, yes = 16/24 present Last dental check-up: <12 months = 17/27 present, ≥12 months = 16/17 present Frequency of professional tooth cleaning: <1/year = 23/26 present, ≥1/year = 10/18 present
	Plaque index (%) (mean ± SD)	Frequency of professional tooth cleaning: <1/year = 73.4 ± 26.5, ≥1/year = 54.4 ± 31.8
	No. of teeth with caries (mean ± SD)	Frequency of professional tooth cleaning: <1/year = 7.2 ± 7.2, ≥1/year = 1.9 ± 2.3
	No. of teeth with PD ≥ 5 mm (Mean ± SD)	Frequency of professional tooth cleaning: <1/year = 6.2 ± 7.7, ≥1/year = 3.5 ± 5.1
**Amin et al. [22]**	Oral hygiene index change (OHI) if brushing	Brushing: no change = 34 (72.3%), change = 13 (27.7%) Not brushing: no change = 5 (83.3%), change = 1 (16.7%); *p* = 1.00
	Oral hygiene index change (OHI) if flossing	Flossing: no change = 6 (85.7%), change = 1 (14.3%) No flossing: no change = 33 (73.0%), change = 13 (27.0%); *p* = 0.81
	Oral hygiene index change (OHI) according to date of last dental visit	<6 months: no change = 3 (73.0%), change = 1 (27.0%) >6 months: no change = 27 (60.0%), change = 6 (40.0%) >1 year: no change = 3 (81.8%), change = 2 (18.2%) Never: no change = 6 (54.6%), change = 5 (45.5%); *p* = 0.22
**Morais et al. [23]**	Oral Assessment Guide (OAG) (mean ± SD)	First exam (post-consent): 10 ± 2.0; second exam (1st RT session): 10 ± 4.0; third exam (15th RT session): 12 ± 2.0 *
	Plaque control record (O’Leary percentage) (mean ± SD)	First exam: 68.0 ± 34.9; second exam: 55.7 ± 37.9 *; third exam: 50.0 ± 39.1 *
	Periodontal screening and recording (PSR) by sextant (mean ± SD)	S1: 1st: 2.0 ± 2.0; 2nd: 1.0 ± 3.0; 3rd: 1.0 ± 3.0 S2: 1st: 2.0 ± 3.0; 2nd: 1.0 ± 2.0 *; 3rd: 0.0 ± 1.0 * S3: 1st: 2.0 ± 2.0; 2nd: 1.0 ± 3.0; 3rd: 1.5 ± 3.0 S4: 1st: 2.0 ± 1.0; 2nd: 1.0 ± 2.0 *; 3rd: 1.0 ± 2.0 S5: 1st: 2.0 ± 1.0; 2nd: 1.0 ± 2.0 *; 3rd: 1.0 ± 1.0 * S6: 1st: 1.0 ± 2.0; 2nd: 0.0 ± 2.0 *; 3rd: 0.0 ± 1.0 *
**Sohn et al. [19]**	Decayed tooth (mean ± SD)	Baseline: healthy group: 0.00 ± 0.00; vulnerable group: 1.29 ± 1.38; *p* = 0.001 * Six-month follow-up: healthy (*n* = 15): 0.00 ± 0.00 → 0.07 ± 0.26, *p* = 0.33; vulnerable (*n* = 14): 1.29 ± 1.38 → 1.57 ± 2.14, *p* = 0.391 Twelve-month follow-up: healthy (*n* = 8): 0.00 ± 0.00 → 0.13 ± 0.354, *p* = 0.351; vulnerable (*n* = 8): 0.88 ± 1.126 → 1.13 ± 1.356, *p* = 0.699
	Plaque index (Loe and Silness) (mean ± SD)	Healthy group: 0.67 ±1.04; vulnerable group: 1.00 ± 0.96; *p* = 0.29 Six-month follow-up: healthy (*n* = 15): 0.66 ± 1.04 → 0.80 ± 1.26, *p* = 0.653; vulnerable (*n* = 14): 1.00 ± 0.96 → 0.92 ± 0.99, *p* = 0.793 Twelve-month follow-up: healthy (*n* = 8): 0.50 ± 1.069 → 0.75 ± 0.707, *p* = 0.451; vulnerable (*n* = 8): 1.00 ± 1.069 → 0.50 ± 0.756, *p* = 0.104
	Gingival index (mean ± SD)	Baseline: healthy group: 1.27 ± 0.96; vulnerable group: 1.43 ± 0.75; *p* = 0.77 Six-month follow-up: healthy (*n* = 15): 1.26 ± 0.96 → 1.40 ± 0.91, *p* = 0.546; vulnerable (*n* = 14): 1.42 ± 0.75 → 1.35 ± 0.92, *p* = 0.793 Twelve-month follow-up: healthy (*n* = 8): 1.25 ± 1.035 → 1.00 ± 1.069, *p* = 0.685; vulnerable (*n* = 8): 1.25 ± 0.886 → 1.50 ± 0.926, *p* = 0.668
	Periodontal pocket depth (mean ± SD)	Baseline: healthy group: 2.67 ± 0.488; vulnerable group: 3.86 ± 1.231; *p* = 0.005 * Six-month follow-up: healthy (*n* = 15): 2.66 ± 0.48 → 2.13 ± 0.74, *p* = 0.041 *; vulnerable (*n* = 14): 3.85 ± 1.23 → 2.92 ± 1.26, *p* = 0.031 * Twelve-month follow-up: healthy (*n* = 8): 2.75 ± 0.463 → 2.38 ± 0.744, *p* = 0.197; vulnerable (*n* = 8): 4.00 ± 0.926 → 2.63 ± 0.518, *p* = 0.008 *
**Frydrych et al. [24]**	Dental caries at cancer diagnosis	Dental attendance: yes = 38, no = 5 (*p* = 0.2676) Dietary advice: yes = 33, no = 10 (*p* = 0.0044 *) Fluoride use: yes = 34, no = 8 (*p* = 0.0138 *) Oral hygiene instruction: yes = 34, no = 8 (*p* = 0.0632)
	Dental caries after cancer treatment	Dental attendance: yes = 14, no = 5 (*p* = 0.0041 *) Dietary advice: yes = 14, no = 5 (*p* = 0.0339 *) Fluoride use: yes = 15, no = 5 (*p* = 0.0183 *) Oral hygiene instruction: yes = 14, no = 6 (*p* = 0.0087 *)
	Dental extractions after treatment	Dental attendance: yes = 18, no = 4 (*p* = 0.0746) Dietary advice: yes = 17, no = 5 (*p* = 0.1284) Fluoride use: yes = 18, no = 5 (*p* = 0.0482 *) Oral hygiene instruction: yes = 18, no = 5 (*p* = 0.1385)
	Development of osteoradionecrosis (ORN)	Dental attendance: yes = 14, no = 2 (*p* = 0.6164) Dietary advice: yes = 13, no = 4 (*p* = 0.0861) Fluoride use: yes = 12, no = 5 (*p* = 0.0127 *) Oral hygiene instruction: yes = 10, no = 7 (*p* = 0.0006 *)

* It means there is statistically significant difference.

**Table 5:** Summary of key oral health outcomes (GI, PI, DMFT) reported across included studies.

Study	Outcome Measure	Reported Results
**Ambati et al. [18]**	Gingival index (mean ± SD)	Baseline: 0.0983 ± 0.21549; 3–6 M: 0.0199 ± 0.06565; 9–12 M: 0.0058 ± 0.03195
	Oral hygiene index (DI-S) (mean ± SD)	Baseline: 1.5820 ± 1.13729; 3–6 M: 1.3350 ± 0.85867; 9–12 M: 1.6213 ± 1.14416
	DMFT (mean ± SD)	Baseline: 3.98 ± 3.93; 3–6 M: 5.03 ± 4.23; 9–12 M: 5.20 ± 3.93
	Plaque score (O’Leary %)	Before: 24.70 ± 22.23; after 6 M: 5.90 ± 5.00; *p* < 0.001
**Lee et al. [20]**	DMFT (mean ± SD)	Before: 5.45 ± 5.64; after 6 M: 5.45 ± 5.77; control: before 6.37 ± 8.12 → 8.40 ± 8.27
	Plaque score (O’Leary %)	Before: 15.74 ± 12.25; after: 21.52 ± 20.61; *p* = 0.113
**Bertl et al. [21]**	Plaque index (%)	<1/year: 73.4 ± 26.5; ≥1/year: 54.4 ± 31.8
	No. of teeth with caries (mean ± SD)	<1/year: 7.2 ± 7.2; ≥1/year: 1.9 ± 2.3
	DMFT (mean ± SD)	Varied by group and timepoint
	Plaque index (Loe and Silness)	Baseline: 1.00 ± 0.96; 12 M: healthy: 0.75, vulnerable: 0.50
**Amin et al. [22]**	Oral hygiene index change	Change rate brushing vs. no brushing; flossing vs. no flossing
**Morais et al. [23]**	Plaque control record (O’Leary %)	1st: 68.0 ± 34.9; 2nd: 55.7 ± 37.9; 3rd: 50.0 ± 39.1
	Periodontal screening and recording (PSR)	Sextant-wise reduction over 3 visits
**Sohn et al. [19]**	Plaque index (Loe and Silness)	Baseline: healthy, 0.67 ± 1.04; vulnerable, 1.00 ± 0.96; NS
	Gingival index	Baseline to 12 M: non-significant changes
	Periodontal pocket depth (mean ± SD)	Baseline: healthy, 2.67; vulnerable, 3.86; ↘ after 6 M and 12 M
	DMFT (dental caries at diagnosis and after)	Fluoride, diet advice, and attendance linked to lower caries
**Frydrych et al. [24]**	Dental caries after cancer treatment	*p* values for reduction in caries across behaviors
	Oral hygiene instruction impact	Instruction linked to improved caries/ORN prevention

## Data Availability

The data presented in this study are available in the article.

## Supplementary Materials

The following supporting information can be downloaded at: https://www.mdpi.com/article/10.3390/diseases13060186/s1, Table S1: Summary of Intervention Characteristics and Oral Health Outcomes Across Included Studies.

## Abbreviations

The following abbreviations are used in this manuscript:

PRISMA	Preferred Reporting Items for Systematic Reviews and Meta-Analyses
PROSPERO	International Prospective Register of Systematic Reviews
GI	Gingival Index
DMFT	Decayed, Missing, and Filled Teeth
RT	Radiotherapy
PBMT	Photobiomodulation Therapy
OHI-S	Simplified Oral Hygiene Index
CPI	Community Periodontal Index
OAG	Oral Assessment Guide
PI	Plaque Index
PD	Probing Depth
ORN	Osteoradionecrosis
SD	Standard Deviation
WMD	Weighted Mean Difference
ROBINS-I	Risk Of Bias In Non-randomized Studies of Interventions
NIH	National Institutes of Health
AXIS	Appraisal Tool for Cross-Sectional Studies
MASCC/ISOO	Multinational Association of Supportive Care in Cancer/International Society of Oral Oncology
EBMT	European Society for Blood and Marrow Transplantation
RevMan	Review Manager
CI	Confidence Interval
