# Supplementary material for: The Impact of Preventive Protocols on Oral Health Outcomes in Cancer Patients Undergoing Chemotherapy or Radiotherapy: A Systematic Review and Meta-Analysis
Source: Diseases. 2025 Jun 16;13(6):186. doi: 10.3390/diseases13060186 (PMC12192284; doi:10.3390/diseases13060186)
Supplement: Supplementary file 1 [file diseases-13-00186-s001.zip › diseases-3658317-supplementary.pdf]

Table S1 Summary of Intervention Characteristics and Oral Health Outcomes Across Included Studies

Study	*Population*	*Hygiene Instruction*	*Fluoride Use*	*PBMT*	*Cancer/Treatment Context*	*Evaluation Timepoints*	*Main Outcomes*	*Preventive Strategy*
Ambati	Children	Standard brushing instruction	1000 ppm toothpaste	No	Pediatric leukemia (chemotherapy)	Baseline, 3-6m, 9-12m	Mucositis, gingival inflammation, caries	Basic education + follow-up
Amin	Adults	Oral hygiene education with leaflets and models	Fluoride gel and varnish (Duraphat)	No	Mixed adult cancers (chemotherapy)	Baseline, 6 weeks	OHI-S, CPI	Hygiene education only
Bertl	Adults	Professional hygiene at least once/year	-	No	HNSCC, post-treatment	Post-treatment only	DMFT, periodontal status	Retrospective grouping by care
Frydrych	Adults	-	5000 ppm toothpaste recommended	No	HNC, post-radiotherapy	Mean follow-up 45m	Compliance, caries	Compliance-focused protocol
Lee	Adults	Oral health education during study visits	-	No	HNC, during/after radiotherapy	Baseline, post-RT, 3m, 6m	Caries, PS, BOP, QoL	Comprehensive oral care
Morais	Adults	Daily hygiene monitoring + education (POCP)	Fluorotherapy (type not specified)	Yes (Daily PBMT in POCP)	HNC, during radiochemotherapy	Baseline, 7th/14th session, end of RT	Mucositis, oral symptoms, QoL, RT interruption	POCP + PBMT
Sohn	Adults	Weekly brushing instruction during RT, then every 3 months	Fluoride varnish (instead of custom tray)	No	HNC, post-radiotherapy	Baseline, 6m, 12m	Caries, plaque, GI, pocket depth	Professional care sessions

BOP – Bleeding on Probing

CPI – Community Periodontal Index

DMFT – Decayed, Missing, and Filled Teeth

GI – Gingival Index

HNC – Head and Neck Cancer

HNSCC – Head and Neck Squamous Cell Carcinoma

OHI-S – Oral Hygiene Index – Simplified

PBMT – Photobiomodulation Therapy

POCP – Preventive Oral Care Protocol

PS – Plaque Score

QoL – Quality of Life

RT – Radiotherapy
